# Sexsomnia: an umbrella review of clinical, neurophysiological and diagnostic evidence

**DOI:** 10.3389/fneur.2026.1795824

**Published:** 2026-05-20

**Authors:** Ioannis Mavroudis, Foivos Petridis, Alin Ciobica, Roxana Cojocariu, Dimitrios Kazis, Ahmed Adel Mansour Kamar, Cătălina Ionescu, Diana Gheban, Catalin Morosan, Bogdan Gurzu, Otilia Novac, Bogdan Novac

**Affiliations:** 1 Leeds Teaching Hospitals, NHS Trust, Leeds, United Kingdom; 2 Romanian Academy of Scientists (Academia Oamenilor de Știință din România), Bucharest, Romania; 3 Third Department of Neurology, Aristotle University of Thessaloniki, Thessaloniki, Greece; 4 Department of Biology, Faculty of Biology, “Alexandru Ioan Cuza” University of Iasi, Iasi, Romania; 5 “Ioan Hăulică” Institute, “Apollonia” University of Iași, Iasi, Romania; 6 "Olga Necrasov" Center, Biomedical Research Group, Romanian Academy, Iasi, Iasi, Romania; 7 CENEMED Platform for Interdisciplinary Research, University of Medicine and Pharmacy “Grigore T. Popa”, Iași, Romania; 8 Department of Biological and Morphological Sciences, Faculty of Medicine and Biological Science, Stefan Cel Mare University of Suceava, Suceava, Romania; 9 GUPCO – Medical Department, Cairo, Egypt; 10 Department of Orthopedics and Traumatology, Clinical Recovery Hospital (Recuperare), Iasi, Romania; 11 Doctoral School of Biology, Faculty of Biology, “Alexandru Ioan Cuza” University of Iași, Iasi, Romania; 12 Faculty of Medicine, Grigore T. Popa University of Medicine and Pharmacy, Iasi, Romania

**Keywords:** cognitive behavioral therapy, differential diagnosis, disorders of arousal, forensic sleep medicine, medicolegal implications, nocturnal sexual automatisms, non-rapid eye movement parasomnia, obstructive sleep apnea

## Abstract

**Background:**

Sexsomnia is a NREM parasomnia involving involuntary sleep-related sexual behaviors that has attracted increasing clinical and forensic attention. However, the existing literature remains fragmented and largely dominated by case reports and small clinical series.

**Objective:**

This umbrella review aims to synthesize review-level evidence by integrating clinical and neurophysiological insights while identifying critical gaps in diagnosis and management.

**Methods:**

Following PRISMA 2020 guidelines, major databases were systematically searched up to January 2025. Systematic, narrative, and scoping reviews were included and assessed using the AMSTAR 2 tool.

**Results:**

Nine reviews were analyzed. Sexsomnia is consistently described as a disorder of arousal emerging from N2/N3 sleep, characterized by sexual automatisms and subsequent amnesia. Neurophysiological findings support a model of state dissociation between motor activation and incomplete cortical awakening. Key triggers include sleep deprivation, alcohol consumption, and obstructive sleep apnea. Diagnostic challenges persist, particularly in differentiating involuntary behaviors from deliberate acts.

**Conclusion:**

Despite recognition as a clinical entity, sexsomnia lacks standardized diagnostic criteria and robust empirical evidence. Future research should focus on establishing consensus diagnostic frameworks and validating objective assessment tools.

## 1 Introduction

Sexsomnia, defined as involuntary sexual behaviors arising during sleep, represents a distinctive and often distressing expression of non-rapid eye movement (NREM) parasomnias. While historically overlooked or dismissed, a growing body of literature has sought to conceptualize sexsomnia within the broader context of sleep medicine. Most notably, contemporary narrative reviews have detailed its clinical presentation—ranging from pelvic thrusting and moaning to complex sexual intercourse, alongside its triggers, neurophysiology, and profound psychosocial impact (1).

Although early literature on sexsomnia was largely based on isolated case reports, several case series have provided broader clinical insight into this condition. Riha et al. described a cohort of 65 patients with sleep-related sexual behaviors, while Muza et al. reported 41 cases and Dubessy et al. analyzed 17 patients. Earlier foundational case series by Guilleminault et al. and Shapiro et al. further supported the classification of sexsomnia as a disorder of arousal arising from NREM sleep. These studies highlight that sexsomnia is not limited to anecdotal reports but represents a reproducible clinical phenomenon within sleep medicine.

Sexual parasomnias encompass a broad spectrum of behaviors, ranging from simple motor acts to complex, goal-directed activities. These include masturbation, rhythmic pelvic thrusting, sexual touching, vocalizations (moaning or explicit verbalizations), ejaculation, and attempts at—or completion of—sexual intercourse (2–5). A defining feature of these episodes is that they occur without conscious awareness or subsequent recall (amnestic episodes). This clinical profile aligns with the ICSD-3-TR classification, which categorizes sexsomnia as a variant of confusional arousals from NREM sleep, under the designation of “sleep-related abnormal sexual behavior” (6).

Evidence from modern studies validates this taxonomy by identifying significant overlaps in both clinical presentation and physiological arousal patterns (3, 4). Historically, the literature from the mid-2000s laid the groundwork for this field by providing comprehensive descriptions of sleep-related sexual activities, consistently framing them as involuntary manifestations of NREM sleep (4, 5).

Epidemiological data suggest a higher prevalence of sexsomnia among male patients, frequently observing it alongside other NREM-related phenomena like somnambulism, night terrors, or confusional arousals (7, 8). These episodes often trigger profound psychological and social repercussions, especially when sleep-related automatisms are mistaken for intentional actions by partners or involve non-consenting parties (9, 10). Consequently, the literature frequently reports heightened levels of psychiatric distress, interpersonal conflict, and significant emotional strain within affected relationships (8).

Extensive literature reviews have pinpointed several precipitating factors for sexsomnia episodes, notably sleep debt, alcohol intake, emotional exhaustion, and inconsistent sleep–wake cycles, as well as the administration of sedative-hypnotic medications (2, 8, 11, 12). Furthermore, clinical reports indicate that obstructive sleep apnea (OSA) may act as a potent physiological trigger; notably, the frequency of these sexual parasomnias often decreases significantly once the underlying respiratory disturbances and subsequent arousals are addressed through appropriate treatment (7).

The neurobiological underpinnings of sexsomnia are typically conceptualized via the ‘state dissociation’ hypothesis. Under this framework, the limbic and motor regions of the brain undergo a sudden transition to wakefulness during sleep, while the prefrontal cortex, the center for executive control and self-awareness, remains in a state of inhibition (2, 13, 14). Such a mechanism allows for the execution of intricate, goal-oriented motor patterns in the absence of conscious volition, a finding that aligns with broader NREM parasomnia studies on the divergence between behavioral output and subjective awareness (15–17).

The identification of sexsomnia is often complicated by the elusive nature of nocturnal sexual behaviors during laboratory monitoring. Diagnosis thus necessitates a multi-modal approach, prioritizing collateral history and the exclusion of intentionality or alternative sleep disorders (3, 12, 18). Currently, the field lacks a standardized screening tool, a deficiency that complicates medicolegal evaluations where the distinction between automatism and deliberate sexual assault is important (19). Given that existing knowledge is distributed across disparate studies, the present umbrella review aims to consolidate high-level evidence regarding the neurophysiological and behavioral dimensions of sexsomnia to clarify ongoing academic and legal controversies.

## 2 Materials and methods

The methodology of this umbrella review was structured in accordance with the PRISMA 2020 (Preferred Reporting Items for Systematic Reviews and Meta-Analyses) guidelines to ensure a transparent and systematic synthesis of existing evidence. Our primary objective was to aggregate and analyze all available review-level literature focusing on sexsomnia and related sexualized behaviors within the spectrum of NREM parasomnias.

To identify relevant literature, we performed a comprehensive search across multiple electronic databases, including PubMed, Embase, PsycINFO, Web of Science, Scopus, and the Cochrane Library, covering all records up to January 2025. The search architecture utilized a combination of keywords targeting sexualized sleep behaviors (e.g., ‘sexsomnia’, ‘sexual parasomnia’, ‘sleep-sex’, or ‘nocturnal sexual automatisms’) cross-referenced with review-specific descriptors such as ‘meta-analysis’, ‘systematic review’, ‘scoping review’, and ‘forensic analysis’. Furthermore, we conducted a manual snowball search by screening the bibliographies of retrieved articles and seminal publications to ensure all eligible evidence was captured.

Studies were eligible for inclusion if they utilized a narrative, systematic, or scoping review methodology, or if they provided thematic clinical and forensic syntheses. Conversely, we excluded primary research limited to isolated case reports or case series lacking a broader review component. Furthermore, editorials, commentaries, correspondence, animal-based research, and publications not explicitly focused on sleep-related sexual behaviors were omitted from the analysis.

Data were extracted independently by a pair of investigators (R.O.C. and I.M), focusing on key domains: epidemiological trends, clinical and neurophysiological profiles, precipitating factors, diagnostic protocols, and medicolegal implications. To ensure scientific rigor, systematic reviews were evaluated using the AMSTAR 2 tool, while narrative syntheses were qualitatively assessed based on their methodological transparency and comprehensiveness. Due to the inherent diversity in study designs and outcome measures, a qualitative narrative synthesis was employed to integrate the findings.

## 3 Results

**Figure 1:**
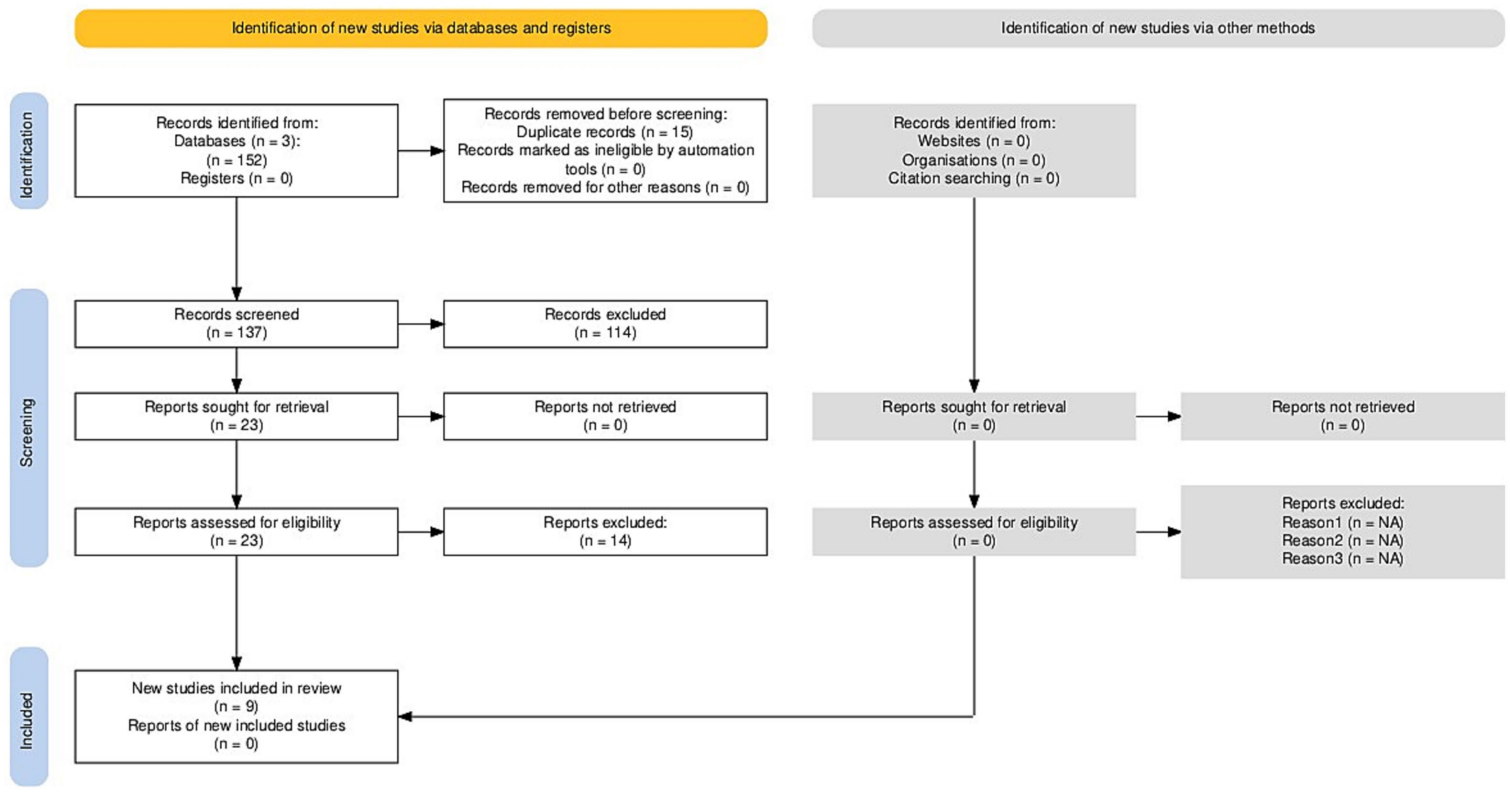
PRISMA flow diagram of the study selection process.

Following a rigorous screening process, nine review-level publications were identified as meeting the predefined inclusion criteria. The selected literature encompasses a diverse range of perspectives, from detailed clinical phenomenologies and neurophysiological frameworks of NREM parasomnias to specialized reviews on behavioral interventions and forensic implications. Despite the methodological diversity of the included studies, they provide a unified characterization of sexsomnia as a distinct clinical entity. This collective evidence highlights its unique symptomatic profile, the role of specific precipitating factors, and the profound challenges it presents within both medical and legal contexts, as shown in Figure 1.

Current research portrays sexsomnia as a distinct manifestation of NREM arousal disorders (6), typically surfacing from N2 or N3 sleep stages (3, 5). The identified behaviors—such as fondling, pelvic thrusting, and complex sexual acts—often mimic volitional conduct, yet the lack of awareness and subsequent memory loss confirm their involuntary nature (2, 5). A key finding across the analyzed reviews is the alignment of sexsomnia with the pathophysiology of sleepwalking rather than waking sexual behavior (3). Furthermore, many studies emphasize the state of cognitive fragmentation experienced by patients, often characterized by severe disorientation when an episode is interrupted by awakening (20).

Epidemiological insights from the analyzed reviews remain constrained by a scarcity of community-based data, yet convergent patterns are evident (21). Sexsomnia is predominantly documented in males and shows high rates of co-occurrence with sleepwalking and other arousal disorders (5, 21). The literature identifies a shared set of provocations, with sleep deprivation, psychological strain, and alcohol intake serving as the most prevalent triggers (3). Several authors also highlighted the mechanistic link between respiratory-induced arousals and sexualized sleep behaviors, noting that the management of obstructive sleep apnea often leads to clinical remission (22). These observations align with a diagnostic model centered on sleep-state instability as the fundamental driver of nocturnal sexual automatisms (Table 1).

**Table 1:** Summary and characteristics of included reviews on sexsomnia and sexual parasomnias.

Review	Type	Focus	Key contributions
Dubessy et al. (2007) (5)	Narrative	Sexual parasomnias	Foundational behavioral description
Schenck et al. (2023) (3)	Narrative	DOA physiology	Neurophysiology and pathophysiology
Tufik et al. (2024) (2)	Narrative	Sexual parasomnias	Classification, diagnosis, clinical impact
Galbiati et al. (2023) (48)	Forensic review	Medicolegal issues	Forensic criteria and legal cases
Leu-Semenescu and Arnulf (2022) (20)	Scoping review	Dream mentation	Awareness, recall, parasomnia cognition
Brás et al. (2023) (13)	Systematic	Behavioral treatments	Evidence for psychological interventions
Almeida et al. (2024) (47)	Case + narrative	CBT-S	First structured CBT-S protocol
Schenck et al. (2009) (44)	Forensic	Early legal framework	Parasomnia in criminal cases
ICSD-3 (2023) (6)	Classification	Diagnostic criteria	Standard diagnostic framework

Neurophysiological insights emerged as a predominant theme across the analyzed reviews, with a consensus framing sexsomnia within the paradigm of state dissociation (19, 23, 24). Under this theoretical model, motor and limbic circuits exhibit wake-like activation during transitions from deep NREM sleep, while higher-order cortical regions—responsible for executive function, inhibitory control, and self-awareness—remain in a state of functional suppression (3, 25). This hybrid neural architecture provides a mechanistic explanation for the execution of intricate sexual automatisms in the absence of conscious volition or memory encoding (5, 19). Furthermore, the literature highlights that this specific dissociated state distinguishes sexsomnia from REM-based parasomnias and the stereotyped motor patterns characteristic of nocturnal frontal lobe epilepsy (3) (Table 2).

**Table 2:** Diagnostic criteria for sexsomnia (ICSD-3 synthesis).

Domain	Description
Behavior	Involuntary sexual acts during sleep
Arousal State	Arises from NREM (N2/N3) sleep
Awareness	Reduced responsiveness during events
Recall	Amnesia or fragmentary recall
Impact	Causes distress, risk, or impairment
Exclusions	RBD, epilepsy, intoxication, intentional behavior

### 3.1 Therapeutic interventions and management

The difficulty of achieving a definitive diagnosis is a central theme throughout the literature, primarily because sexual automatisms are seldom recorded during polysomnography. Consequently, clinicians must prioritize a comprehensive medical history and the testimony of bed partners to establish a diagnosis (26). The reviews highlight the necessity of a meticulous differential diagnosis to rule out confounding conditions such as REM sleep behavior disorder (RBD), nocturnal frontal lobe epilepsy, and substance-induced manifestations (6, 27). In the forensic arena, these diagnostic standards are even more critical, as sexsomnia is frequently invoked as a legal defense to contest criminal responsibility in sexual assault cases. Authors warn of the high stakes involved in misclassification, urging practitioners to maintain rigorous evaluative protocols when assessing such medicolegal claims (28) (Table 3).

**Table 3:** Differential diagnosis.

Feature	Sexsomnia	RBD	Nocturnal hypermotor epilepsy
Sleep stage	N2/N3	REM	N2
Recall	Absent	Dream recall	Absent
Behavior	Sexual automatisms	Dream enactment	Stereotyped hypermotor
Duration	1–15 min	0.5–5 min	Seconds–2 min
PSG findings	Arousal from slow-wave sleep	REM without atonia	Epileptiform activity
Key clues	Parasomnia history	Older age	Clustered, stereotyped events

Current literature regarding the treatment of sexsomnia, though limited, suggests a multi-modal approach centered on stabilizing sleep architecture. Most reviews prioritize behavioral strategies designed to mitigate sleep-state instability, such as maintaining consistent sleep–wake cycles, abstaining from alcohol, and managing psychological stressors (3). A significant emphasis is placed on the resolution of comorbid sleep disorders, particularly obstructive sleep apnea (OSA); evidence indicates that managing respiratory-induced arousals can lead to the partial or complete remission of sexualized behaviors (22). Recent systematic and narrative reviews provide preliminary support for cognitive-behavioral interventions (CBT-I), with emerging research detailing specific protocols, such as CBT-S, tailored for sexual parasomnias (13). Conversely, pharmacological evidence remains largely anecdotal and sparse, with only isolated reports suggesting potential benefits from agents such as paroxetine and clonazepam, with clonazepam showing reported improvement in the majority of patients in small case series childhood parasomnias or comorbid adult parasomnia (3, 29).

Sexsomnia has important forensic implications, particularly when behaviors are misinterpreted as intentional acts. In such cases, multidisciplinary evaluation involving sleep specialists, psychiatrists, and legal experts is essential. Current developments in forensic sleep medicine emphasize structured assessment approaches, including objective sleep investigations, collateral history, and careful exclusion of alternative explanations such as epilepsy, intoxication, or malingering (30) (Table 4).

**Table 4:** Thematic coverage of reviews.

Review	Class	Phenomenology	Neurophysiology	Treatment	Forensic
Dubessy 2007 (4)	✓	✓✓✓	✓	✓	✓
Schenck 2023 (2)	✓✓	✓✓	✓✓✓	✓	—
Tufik 2023 (1)	✓	✓✓✓	✓✓	✓	✓
Rinaldi 2023 (5)	✓	✓	✓	—	✓✓✓
LS and Arnulf 2022	—	✓	✓✓	—	—
Pinto 2023 (7)	—	✓	✓	✓✓✓	—
Almeida 2024 (6)	—	✓	—	✓✓	—

## 4 Proposed diagnostic evaluation framework for sexsomnia

**Figure 2:**
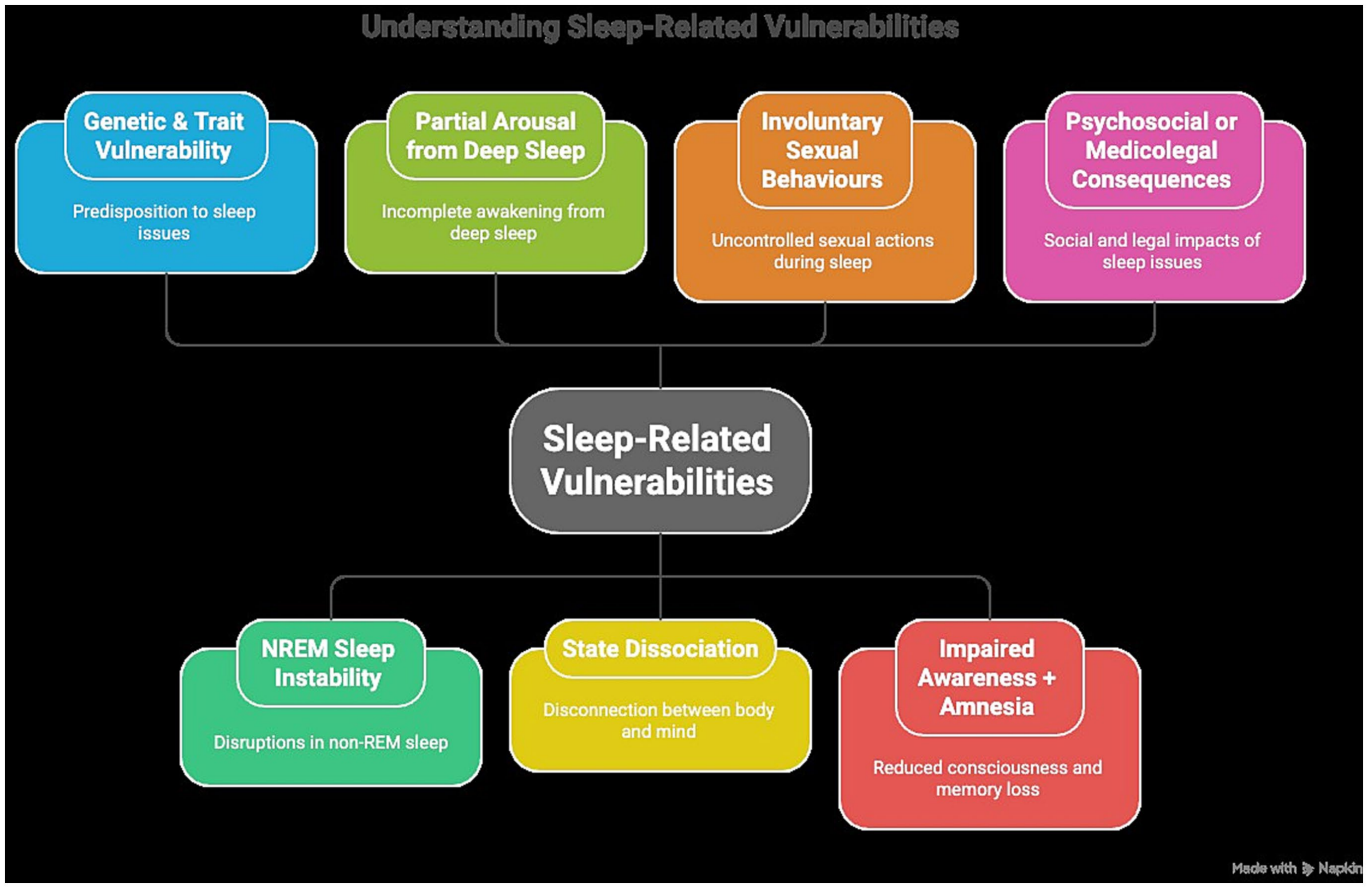
Conceptual framework of sleep-related vulnerabilities in sexsomnia pathophysiology. Flowchart illustrating the stepwise evaluation of suspected sexsomnia, including differential diagnosis and classification into probable, atypical, and alternative cases.

While a universally standardized diagnostic protocol for sexsomnia has yet to be formally established, the consensus emerging from the analyzed review literature offers a robust foundation for an evidence-based clinical evaluation framework. The development of such a framework is critical, particularly given the inherent challenges in capturing nocturnal sexual events via polysomnography, the complexity of differential diagnosis, and the escalating forensic implications associated with the disorder. By synthesizing the clinical, neurophysiological, and medicolegal insights gathered in this umbrella review, it is possible to articulate a multidimensional assessment strategy designed to aid practitioners in establishing a reliable diagnosis of sexsomnia (Figure 2).

Video-polysomnography (vPSG) remains the gold standard in cases of diagnostic uncertainty, particularly when distinguishing sexsomnia from other complex nocturnal behaviors. While vPSG findings in sexsomnia are often nonspecific and rarely capture the sexual automatisms themselves, they can provide critical information regarding sleep architecture, arousal patterns, and the exclusion of epileptiform activity. Complementary investigations such as EEG and neuroimaging are essential when structural lesions or sleep-related hypermotor epilepsy (SHE) are suspected, ensuring a comprehensive differential diagnostic approach.

The initial phase of evaluating suspected sexsomnia must involve a meticulous analysis of the behavioral phenomenology. The analyzed literature consistently characterizes these episodes as non-volitional sexual automatisms that emerge during N2 or N3 sleep stages, defined by a significant impairment of consciousness, lack of responsiveness to external stimuli, and subsequent amnesia. These diagnostic markers are fundamental, as they differentiate involuntary nocturnal acts from deliberate, wakeful sexual conduct.

Given that patients often have no subjective recollection of their actions, obtaining collateral history from bed partners or witnesses is indispensable. Witness testimony is critical for verifying the automatic nature of the behavior, observing signs of postictal confusion or disorientation, and noting a rapid return to sleep—all of which are hallmark features of NREM-related disorders of arousal.

A second critical dimension of the diagnostic process involves evaluating the patient within the broader clinical framework of NREM parasomnias. Research consistently indicates that sexsomnia often coexists with other disorders of arousal, including sleepwalking or night terrors. The likelihood of a sexsomnia diagnosis is significantly increased if there is a personal history of childhood parasomnias, a family history of similar conditions, or current evidence of confusional arousals (29, 31).

Furthermore, the evaluation should identify potential precipitating factors that exacerbate NREM instability, such as sleep deprivation, shift work, psychological strain, alcohol intake, or the use of sedative-hypnotic medications (32, 33). It is also essential to screen for obstructive sleep apnea (OSA), as literature suggests that treating respiratory-induced arousals can lead to the successful resolution of sexualized sleep behaviors.

**Figure 3:**
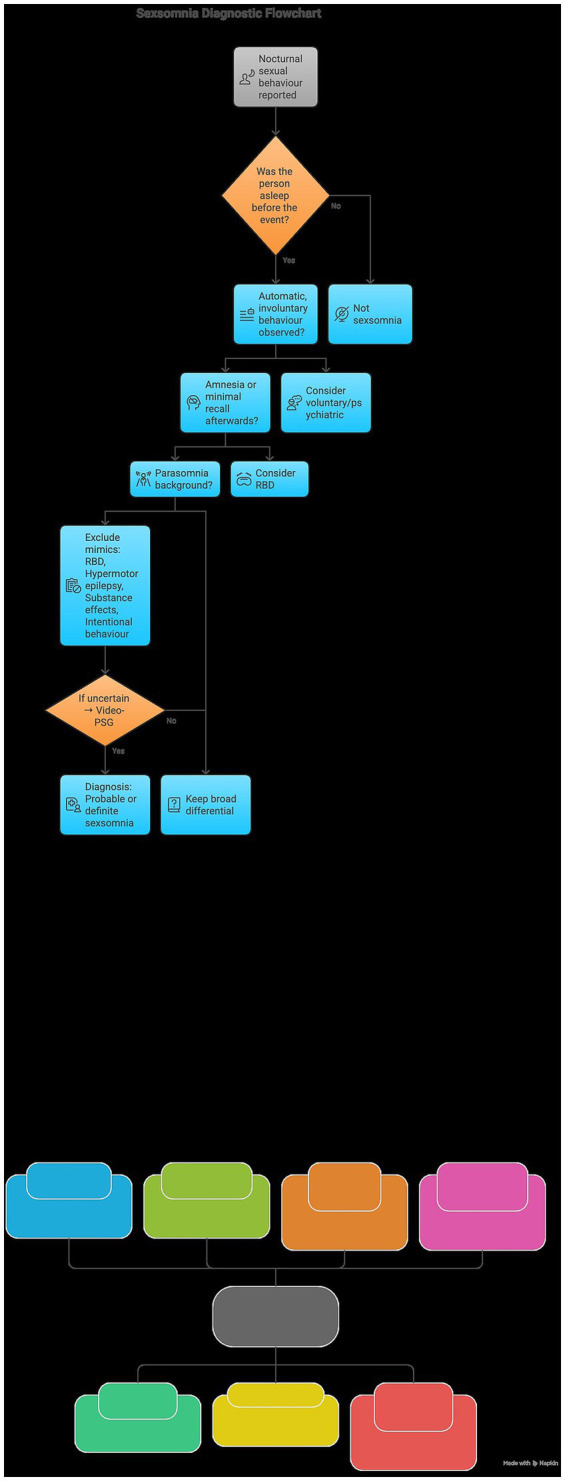
Diagnostic pathway for sexsomnia and differential screening.

A critical phase of the clinical evaluation involves a systematic differential diagnosis to distinguish sexsomnia from other sleep-related disturbances. Practitioners must differentiate these sexualized behaviors from conditions with overlapping clinical presentations. For instance, REM sleep behavior disorder (RBD) is defined by the physical enactment of dreams during the REM stage, usually accompanied by vivid recall and a characteristic loss of muscle atonia (34). In contrast, nocturnal hypermotor epilepsy often manifests as abrupt, repetitive, and stereotyped motor discharges; these episodes are typically shorter than sexsomnia events and may show specific ictal changes on an EEG (35) (Figure 3).

Furthermore, behaviors induced by substances or alcohol, particularly sedative-hypnotic agents, can simulate parasomnia-like activity. However, these are generally linked to acute intoxication and do not strictly align with the physiological transitions of NREM sleep (36, 37). In medicolegal contexts, the possibility of malingering or deliberate conduct must be rigorously investigated to ensure the integrity of the diagnostic conclusion. Should diagnostic ambiguity remain, the use of video-polysomnography (vPSG) for extended monitoring is recommended, although the existing literature cautions that such studies rarely capture the actual sexual automatisms.

A fourth dimension of the assessment involves a thorough examination of psychological and relational dynamics. While sexsomnia is not classified as a psychiatric illness, the literature frequently identifies significant associations with psychological distress, interpersonal strain, and profound feelings of shame or conflict stemming from the episodes (38). Analyzing the psychosocial context is essential to determine if behaviors align with typical parasomnia patterns or if they point toward alternative etiologies. It is important to note that the presence of psychiatric comorbidities does not preclude a sexsomnia diagnosis, but it does necessitate a more nuanced and careful evaluation (39).

Finally, forensic considerations must be meticulously integrated into the evaluation when relevant. In legal cases where sleep-related sexual behaviors result in alleged harm, the clinical assessment must be exceptionally comprehensive, rigorously documented, and rooted in established sleep science. The credibility of a sexsomnia diagnosis in a medicolegal context is reinforced by a documented history of parasomnias, consistent witness testimony, identified sleep instability or triggers, and the absence of contradictory behavioral evidence. Conversely, practitioners must maintain a high level of scrutiny regarding inconsistencies between the reported actions and known parasomnia phenomenology, the presence of situational motives, or a lack of external corroboration (25, 31).

Taken together, these components establish a robust diagnostic evaluation network: a multi-layered assessment process that integrates behavioral phenomenology, the clinical context of NREM parasomnias, rigorous differential diagnosis, psychosocial evaluation, and forensic analysis when indicated. Although this framework does not yet constitute a formalized diagnostic criterion in the vein of traditional psychiatric classifications, it effectively synthesizes convergent evidence from current literature to provide clinicians with a structured model for investigating suspected sexsomnia. The development of this model highlights the urgent need for future research to validate specific diagnostic markers and to create standardized assessment tools capable of distinguishing sexsomnia from its mimics with greater reliability. Based on the integrated clinical and paraclinical findings, patients can be categorized into three diagnostic tiers: “probable sexsomnia” (typical NREM parasomnia pattern with amnesia and collateral confirmation), “atypical cases” (mixed or inconclusive features requiring further investigation), and “alternative diagnosis” (clear evidence of other conditions such as epilepsy, REM sleep behavior disorder, or substance-induced behaviors) (Table 5).

**Table 5:** Multi-domain diagnostic framework and clinical checklist for the evaluation of suspected sexsomnia.

Assessment domain	Key diagnostic criteria and observation points
1. Collateral history (partner/witness)	Sleep onset: Did the behavior emerge while the patient was clearly asleep? Phenomenology: Specific actions observed (masturbation, fondling, intercourse attempts, sexual vocalizations)? Responsiveness: Level of impaired awareness or ability to follow instructions? Ocular signs: Eyes open/closed, presence of a “blank stare,” or lack of tracking? Temporal pattern: Duration, frequency, and timing (first, middle, or last third of night)? Consequences: Physical injuries to self or the partner? Associated DOA: Concurrent sleepwalking, terrors, or confusional wandering?
2. Patient subjective history	Amnesia: Degree of recall (none, partial, or full)? Mentation: If recall exists, presence of dream content (vague vs. vivid/sexual)? Predisposition: History of childhood NREM parasomnias or family history? Triggers: Sleep deprivation, irregular schedules, binge alcohol use, or stress? Pharmacology: Use of Z-drugs, sedatives, or new antidepressants?
3. Screening for mimics	RBD: Age >50, vivid dream enactment, or non-sexual acting out (fighting/shouting)? Epilepsy (SHE/NFLE): Brief (<2 min), stereotyped, clustered episodes; history of daytime seizures? Substance effects: Behaviors occurring solely during acute intoxication or after specific medication doses? Voluntary/forensic: Discrepancies between amnesia and digital evidence; clear external legal gain?
4. Physical exam and diagnostics	Neurological/mental exam: Assessment for focal signs, cognitive impairment, or dissociation? Video-PSG (vPSG): Indicated for diagnostic doubt, atypical onset, or high-stakes legal cases? Ancillary: EEG and imaging if epilepsy or structural brain lesions are suspected?
5. Provisional formulation	Probable sexsomnia: Classic NREM pattern, amnesia, and established DOA history. Atypical: Mixed features; requires further PSG or EEG data before confirmation. Alternative diagnosis: Clear evidence of epilepsy, RBD, or volitional behavior; manage accordingly.

## 5 Discussion

This umbrella review provides a comprehensive synthesis of current literature on sexsomnia, identifying it as a distinct clinical entity firmly rooted in the spectrum of non-rapid eye movement (NREM) parasomnias. Although sexsomnia has gained prominence in both medical and forensic circles, the current evidence base is largely dominated by narrative overviews and individual case studies rather than large-scale empirical trials (3, 39). Nevertheless, the cross-study consensus regarding its phenomenology and neurobiological underpinnings allows for a structured understanding of its diagnostic and medicolegal complexities.

Despite increasing recognition of sexsomnia, significant gaps remain in diagnostic standardization. Currently, there are no validated sexsomnia-specific polysomnographic criteria, no standardized vPSG scoring protocol for sexual behaviors, and no consensus regarding the optimal duration of recording. Additionally, no validated clinical interview or rating scale exists for sleep-related sexual behaviors. These limitations contribute to diagnostic uncertainty and increase the risk of misclassification, particularly in medicolegal contexts.

A primary finding of this synthesis is the classification of sexsomnia as a “disorder of arousal” typically originating during N2 or slow-wave sleep (N3). The reported behavioral range—encompassing masturbation, vocalizations, pelvic thrusting, and attempted intercourse—mirrors the core features of other NREM parasomnias like somnambulism (19). These episodes are defined by profound state dissociation, where motor systems are activated while cortical regions responsible for executive function and memory remains dormant, resulting in characteristic amnesia and lack of volitional intent (40, 41). While these actions may appear purposeful to an observer, they are fundamentally automatic and unplanned, occurring within the fragile transition between deep sleep and partial awakening (3, 31). Consequently, sexsomnia is best understood as a specialized phenotypic expression of the broader arousal disorder framework.

The mechanisms underlying sexsomnia are best understood through neurophysiological models of state dissociation. The literature describes this phenomenon as a localized failure of the brain to transition uniformly between sleep and wakefulness (11). During an episode, neural circuits responsible for motor output and emotional processing (the limbic system) become active, while the prefrontal and association cortices—areas governing executive function, judgment, and conscious awareness—remain in a state of inhibition (41). This selective activation explains how complex, seemingly goal-oriented sexual behaviors can occur without volitional control or subsequent memory.

Emerging neurophysiological research suggests that alterations in sleep-dependent coordination mechanisms, such as ripple–spindle coupling, may provide future biomarkers for differentiating parasomnias from epileptic disorders. In particular, the disruption of coordinated hippocampo-cortical activity (e.g., ripple–spindle decoupling) has been proposed as a potential marker distinguishing disorders of arousal from conditions such as sleep-related hypermotor epilepsy (SHE). Although these approaches remain experimental, they represent a promising direction for improving diagnostic specificity in complex nocturnal behaviors.

This model provides a clear biological distinction between sexsomnia and REM sleep behavior disorder (RBD). While sexsomnia involves a dissociation during NREM sleep (N2/N3) and typically lacks dream mentation, RBD is characterized by the failure of muscle atonia during REM sleep, leading to the physical enactment of vivid dreams (42).

Despite these clear theoretical frameworks, clinical diagnosis remains difficult due to the limitations of video-polysomnography (vPSG). The literature consistently notes that sexual automatisms are rarely captured in a laboratory setting (43). When recorded, the results often show non-specific abrupt arousals from slow-wave sleep rather than definitive sexual behaviors or epileptiform discharges (3). Consequently, clinicians must rely on a “diagnostic network” that prioritizes meticulous clinical interviews and collateral history from bed partners to verify the automaticity and postictal confusion characteristic of the disorder (31).

Differential diagnosis is paramount, as sexsomnia must be distinguished from nocturnal hypermotor epilepsy, substance-induced behaviors, and deliberate sexual conduct (34). Reviewers emphasize that a failure to integrate sleep architecture analysis with an evaluation of the patient’s neurological, pharmacological, and psychological profile significantly increases the risk of misdiagnosis, particularly in forensic settings (32).

A major barrier to accurate diagnosis is the absence of standardized diagnostic instruments or structured interviews specific to sexsomnia. While generic parasomnia questionnaires exist, none have been validated for detecting sleep-related sexual behaviors or distinguishing them from mimicking conditions. This gap limits both research and clinical practice and underscores the need for consensus-based diagnostic criteria or validated rating scales tailored to sexsomnia.

The epidemiological landscape of sexsomnia is currently defined by a lack of large-scale, population-based data. Most existing prevalence estimates are derived from specialized sleep clinics, retrospective analyses, and clinical case series (43). While these sources indicate a higher frequency among males and a significant overlap with other NREM parasomnias, the actual prevalence in the general population is likely obscured by widespread underreporting. Patients often fail to seek help due to profound embarrassment, fear of social stigma, or potential legal ramifications (22, 39). Furthermore, research suggests a “parasomnia vulnerability profile,” where sexsomnia is more common in individuals with a genetic predisposition to arousal disorders or a childhood history of somnambulism (41).

The literature consistently identifies a trigger profile for sexsomnia that is identical to that of other arousal disorders. Common precipitants include sleep deprivation, alcohol consumption, high psychological stress, and the use of sedative-hypnotic medications (32). Notably, several clinical reports have established a critical link between sexsomnia and obstructive sleep apnea (OSA). In these cases, respiratory events act as fragmenting stimuli that trigger sleep-related behaviors, many of which resolve upon the initiation of Continuous Positive Airway Pressure (CPAP) therapy (44, 45) or mandibular advancement device therapy (45, 46).

The primary focus remains on non-pharmacological interventions, such as improving sleep hygiene, maintaining consistent sleep schedules, and eliminating known triggers like alcohol (40). When an underlying condition such as OSA is identified, treating the respiratory disorder is often the most effective path to resolution (43). Pharmacological evidence remains anecdotal; while some reviews mention the use of benzodiazepines (like clonazepam) or certain antidepressants, these are not supported by controlled clinical trials and should be approached with caution (3, 39).

Emerging evidence suggests that specialized behavioral approaches, specifically cognitive-behavioral therapy for parasomnias (CBT-P) and its sexsomnia-specific adaptations (CBT-S), represent the most promising therapeutic frontier. These protocols typically target sleep instability by addressing stimulus control, stress management, and the restructuring of maladaptive sleep-related beliefs (40). While preliminary case reports indicate that CBT-S may be particularly effective for patients whose symptoms are exacerbated by psychological distress, the literature currently lacks formal clinical trials to validate its efficacy (39). Consequently, establishing standardized outcome measures for these behavioral interventions remains a critical priority for future study.

Perhaps the most significant dimension of sexsomnia identified in this review is its medicolegal impact. Because these behaviors can appear intentional to an observer, they frequently result in severe interpersonal conflict and legal allegations of sexual misconduct. Forensic analyses highlight the extreme difficulty of assessing “voluntariness” and “intent” when complex actions occur during state dissociation (31). To protect the integrity of the judicial process and prevent the misuse of sexsomnia as an exculpatory claim, courts demand rigorous diagnostic evidence. This includes a verifiable history of NREM parasomnias, consistent witness testimony, and the exclusion of malingering through expert clinical evaluation (25).

The findings of this umbrella review reveal a paradox: while sexsomnia is conceptually well-defined within neurophysiology, it remains empirically under-supported. The field is currently limited by a lack of large-scale prevalence data, validated diagnostic instruments, and controlled therapeutic trials. Furthermore, the absence of specific neurobiological biomarkers makes it difficult to distinguish sexsomnia from its mimics with absolute certainty.

To move the field forward, a coordinated research agenda should prioritize the following: epidemiology—establishing population-based data to identify genetic and demographic risk factors, standardization—developing consensus-based diagnostic criteria and assessment tools, clinical trials—conducting rigorous studies on both pharmacological and behavioral (CBT-S) treatments, advanced neurophysiology—utilizing high-density EEG or functional imaging to identify the specific signatures of sexsomnic arousals and forensic guidelines—creating structured protocols for expert testimony in legal settings.

## 6 Conclusion

Although sexsomnia is a legitimate NREM parasomnia with a clear neurophysiological basis, current evidence is insufficient for standardized clinical or legal practice. Bridging these gaps is essential for improving patient outcomes and ensuring that medicolegal evaluations are grounded in robust sleep science.

## Data Availability

The original contributions presented in the study are included in the article/supplementary material, further inquiries can be directed to the corresponding authors.
